# Supplementary material for: Plant-Derived Molecule 4-Methylumbelliferone Suppresses FcεRI-Mediated Mast Cell Activation and Allergic Inflammation
Source: Molecules. 2022 Feb 27;27(5):1577. doi: 10.3390/molecules27051577 (PMC8912031; doi:10.3390/molecules27051577)
Supplement: Supplementary file 1 [file molecules-27-01577-s001.zip › Table S1 Sequences of primers used in real-time RT-qPCR.pdf]

Table S1. Sequences of primers used in real-time RT-qPCR.

Gene	Forward (5′-3′)	Reverse (5′-3′)
Rat Gapdh	GGCACAGTCAAGGCTGAGAATG	ATGGTGGTGAAGACGCCAGTA
Rat IL-1β	GGGATGATGACGACCTGCTA	TGTCGTTGCTTGTCTCTCCT
Rat Tnfα	CCCTGTTCTGCTTTCTCA	GTTCTCCGTGGTGTTCCT
Rat Il4	GAGGACCAGAACGAGACA	CCAGAAGCGTGACAGAGA
Rat Fcer1A	GGCTGCTGCTCCAATCTTC	GCAATGTCGTCCTTGTAGTAGA
Rat Ms4a2	TGCTCCACACTCCAGACTTC	GCTGCCTCTCACCAGATACA
Rat Fcer1G	GGTGATCTTGTTCTTGCTCCTT	TCACGGCTGGCTATGTCTG
Mouse Gapdh	AAGAAGGTGGTGAAGCAGG	GAAGGTGGAAGAGTGGGAGT
Mouse Il4	GCTAGTTGTCATCCTGCTCTTC	GGTGTTCTTCGTTGCTGTGA
Mouse Il6	TCACAGAAGGAGTGGCTAAGGACC	ACGCACTAGGTTTGCCGAGTAGAT
Mouse IL-1β	TGTGTTTTCCTCCTTGCCTCTGAT	TGCTGCCTAATGTCCCCTTGAAT
Mouse Fcer1A	CCGTCTCTGAGGTGAACTCTT	CAGCCAATCTTGCGTTACATTC
Mouse Ms4a2	TGTAGAAGTCTGGATGGTGGAA	CTAAGTGTAGGCATGTGGAGTT
Mouse Fcer1G	TGCTTTGAAGGTTGGCTGAC	AAGGAGGCTGGAAGAAGAGAA
