# Supplementary figures and images for: Plant-Derived Molecule 4-Methylumbelliferone Suppresses FcεRI-Mediated Mast Cell Activation and Allergic Inflammation
Source: Molecules. 2022 Feb 27;27(5):1577. doi: 10.3390/molecules27051577 (PMC8912031; doi:10.3390/molecules27051577)

# Supplementary Figure S1

a

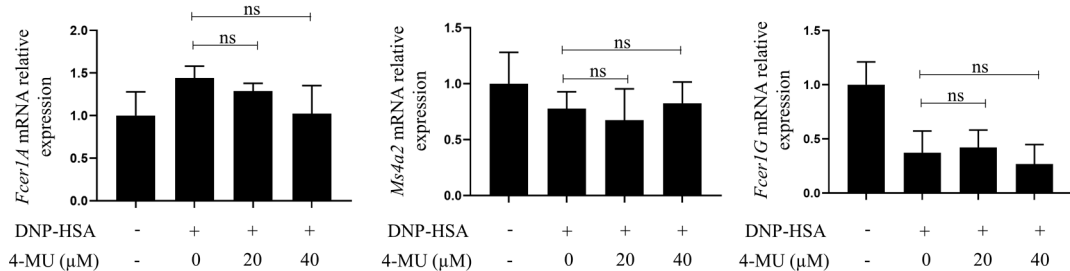

b

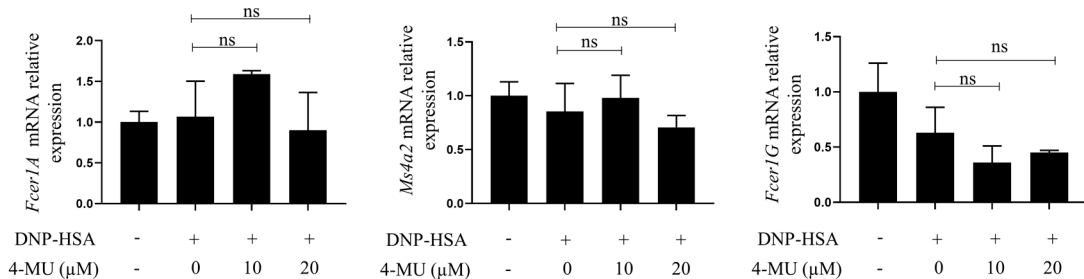

Supplement: Supplementary file 1 [file molecules-27-01577-s001.zip › Figure S1 The cell surface FcRI receptor genes in mast cells is not changed by 4-MU treatment.pdf]
